# Altered gut microbiome in convalescent patients with coronavirus disease 2019

**DOI:** 10.3389/fcimb.2024.1455295

**Published:** 2024-11-28

**Authors:** Kyoung Hwa Lee, Yeong Ouk Kim, So Hee Dho, Jen J. H. Yong, Hyun-Seok Oh, Je Hee Lee, Seung-Jo Yang, Inseong Cha, Jongsik Chun, Eun Hwa Lee, Su Jin Jeong, Wonjin Woo, Jae-Phil Choi, Sang Hoon Han, Gloria B. Choi, Jun R. Huh, Lark Kyun Kim, Young Goo Song

**Affiliations:** ^1^ Division of Infectious Diseases, Department of Internal Medicine, Yonsei University College of Medicine, Seoul, Republic of Korea; ^2^ CJ Bioscience, Inc., Seoul, Republic of Korea; ^3^ Interdisciplinary Program in Bioinformatics, Seoul National University, Seoul, Republic of Korea; ^4^ Department of Biomedical Sciences, Graduate School of Medical Science, Brain Korea 21 Project, Gangnam Severance Hospital, Yonsei University College of Medicine, Seoul, Republic of Korea; ^5^ Department of Immunology, Blavatnik Institute, Harvard Medical School, Boston, MA, United States; ^6^ Division of Infectious Diseases, Department of Internal Medicine, Seoul Medical Center, Seoul, Republic of Korea; ^7^ The Picower Institute for Learning and Memory, Department of Brain and Cognitive Sciences, Massachusetts Institute of Technology, Cambridge, MA, United States; ^8^ Bio2Q, Keio University, Tokyo, Japan

**Keywords:** stool microbiome, metagenomics, SARS-CoV-2, severity of coronavirus disease 2019 (COVID-19), convalescent patients

## Abstract

**Introduction:**

Coronavirus disease 2019 (COVID-19) alters the gut microbiome. This study aimed to assess the association between the disease severity of COVID-19 and changes in stool microbes through a seven-month follow-up of stool collection.

**Methods:**

We conducted a multicentre, prospective longitudinal study of 58 COVID-19 patients and 116 uninfected controls. Differences in the gut microbiota were analysed using 16S ribosomal RNA sequencing. The first stool samples were collected at an early convalescent phase of COVID-19, and the second sample was collected at least seven months after COVID-19 infection.

**Results and discussion:**

At the order level, *Eubacteriales* and *Bifidobacteriales* decreased, while *Bacteroidales* and *Burkholderiales* increased in the COVID-19 group compared to the controls. Alpha diversity also decreased in COVID-19 patients compared to controls, with imperfect recovery of the gut microbiome after seven months. The compositional change in the gut microbiome between the early and late convalescent phases was largest in the moderate and severe groups. The severity of COVID-19 was the most influential clinical variable for microbiome composition (Sum of Sqs = 0.686, *P* = 0.006), and its effect persisted even after partialling out other effects such as antibiotic use and age. Thus, our study indicates a possible interaction between respiratory viral infection and the composition of the gut microbiota community, warranting future mechanistic and prospective longitudinal studies. Additionally, we were able to detect microbiome changes in patients who were re-infected with SARS-CoV-2. Notably, the dominant bacteria in the re-infected group were *Lachnospiraceae* and *Faecalimonas umbilicata*, compared to the one-time infected group.

## Introduction

Since the World Health Organization (WHO) declared a pandemic in March 2020, the threat of Coronavirus disease 2019 (COVID-19) has persisted as variants of Severe Acute Respiratory Syndrome Coronavirus 2 (SARS-CoV-2) continuously emerge (Karim and Karim, 2021). Angiotensin-converting enzyme 2 (ACE2) receptor play a key role in the entry of SARS-CoV-2 into host cells. The spike protein of SARS-CoV-2 binds to the ACE2 receptor, forming a complex that enters and replicates in human cells (Jia et al., 2005; Du et al., 2009; Wang et al., 2020). ACE2 receptors are present in the epithelial cells of multiple tissues, including goblet cells of the nasal cavity, alveolar cells of the lung, mucosal cells of the gastrointestinal tract, tubular epithelial cells of the kidney, and endothelial cells of blood vessels. Therefore, COVID-19 patients may experience various gastrointestinal symptoms, such as vomiting and diarrhea (Dai et al., 2020; Han et al., 2020; Sungnak et al., 2020).

Commensal microbes have been suggested to play critical roles in modulating natural resistance to viral infection in preclinical mouse models (Stefan et al., 2020). Alterations in the gut microbiome communities of patients with COVID-19 are thought to be associated with pneumonia (Dhar and Mohanty, 2020; Gu et al., 2020; Zuo et al., 2020a; Zuo et al., 2020b; Sun et al., 2022).

Bacterial dysbiosis is sustained after COVID-19 resolution and is correlated with an increased incidence of opportunistic pathogens (Yamamoto et al., 2021; Zuo et al., 2021). However, since stool samples for these studies were collected during the early infection period, it is unclear whether COVID-19 is associated with long-term microbiome changes (Chen et al., 2022; Liu et al., 2022). Thus, prolonged dysbiosis in post-COVID-19 patient groups and its correlation with disease severity should also be evaluated. Furthermore, antibiotic use also needs to be considered for its potential confounding effects on gut dysbiosis because most severe COVID-19 patients are exposed to antimicrobial agents due to secondary bacterial pneumonia during hospitalization. Additionally, since the number of re-infection cases is increasing, it is informative to assess the differences in the gut microbiome in those infected once versus twice.

Herein, we compared gut microbiota composition at the early convalescent and late recovery phases after confirming SARS-CoV-2 infection, considering other clinical factors such as antibiotic effects and disease severity. We verified the association between disease severity and stool microbiome alterations in COVID-19 patients and assessed the recovery of gut dysbiosis through a seven-month follow-up stool collection. Additionally, we attempted to identify the dominant bacterial species in patients infected with SARS-CoV-2 once versus twice.

## Materials and methods

### Study design and populations

The enrolment criteria were persons who recovered with improvement of symptoms after being diagnosed with COVID-19 by PCR test, regardless of clinical symptoms. The exclusion criteria were 1) pregnant women, 2) illiterate, and 3) those who did not consent to participate in the study.

Patients were confirmed to have COVID-19 infection by PCR tests using nasopharyngeal and oropharynx swab specimens. This test was performed by RT-PCR using Power Check TM (Kogene Biothech, Republic of Korea), which has been authorized by the Food and Drug Administration (FDA). The threshold was 38 cycles of PCR, thus, with Ct values greater than 38 being treated as negative and Ct values smaller than 37 as positive for infection. Ct values between 37 and 38 were determined as intermediate and PCR was repeated. The PCR test targeted the Envelope (E) gene and RNA-dependent RNA polymerase gene (RdRp) gene of SARS-CoV-2. These tests in this study were performed at three different medical centers from July 2020 to Feb 2022. Sinchon and Gangnam Severance Hospitals are university-affiliated tertiary hospitals with a total of 2,800 beds. The Seoul Medical Center is a public hospital with 500 beds in Seoul, Republic of Korea.

Initial stool samples were collected from the subjects at the end of quarantine or hospitalization. Hospitalization and isolation are separate issues. If a patient was admitted and confirmed with COVID-19, they were moved to a negative pressure isolation facility and quarantined. Usually, if the confirmed COVID-19 status had been known before admission, the date of admission and the start date of isolation were determined the same. The quarantine is maintained for 7 days from the date of confirmation, and once it ends, patients were moved out of the negative pressure isolation facility and into a general ward. Follow-up stool samples were collected via the express mail system seven months after the date of COVID-19 confirmation. For the freshness of stool samples, the patients were limited to local residents within 60 km from the laboratory of this hospital, who could deliver stool specimens to the research lab within 2 hours after defecation. If the patients had mild symptoms and were discharged with brief hospitalization within seven days, stool samples were collected within one month of the diagnosis of COVID-19.

### Healthy SARS-CoV-2 naive controls

We compared the gut microbiome of early and late convalescent phases of COVID-19 patients with healthy controls (1:2 ratio). Propensity score matching analysis was used based on three variables: age, sex, and body mass index (BMI). The data for the healthy control group were provided by the data platform of CJ Bioscience, Inc. (Seoul, South Korea) for comparative analysis. Healthy individuals were selected from gut microbiota study of healthy soldiers in the Republic of Korea (Yoon et al., 2021). The exclusion criteria included history of acute illness, chronic illness, cancer, abdominal surgery, and the use of antibiotics or probiotics within 3 months of the study. For individuals older than 60 years, the exclusion criteria for the presence of hypertension and diabetes were dismissed if they were well controlled. All healthy control samples were collected prior to the COVID-19 pandemic.

### Data collection and clinical variables

Clinical characteristics, including age, sex, height, body weight, vaccination, smoking and clinical symptoms during hospitalization, and underlying medical conditions, were investigated using self-report questionnaires. The severity of disease was stratified as mild (severity score, l; no limitation of activities, and 2; limitation of activities), moderate (3: hospitalized with no oxygen therapy, and 4: oxygen therapy by mask or nasal prongs), and severe (5; non-invasive mechanical ventilation or high-flow oxygen therapy, 6; mechanical ventilation; and 7; mechanical ventilation with renal replacement therapy or Extracorporeal Membrane Oxygenation) by the WHO ordinal severity scale based on the clinical and respiratory status of patients (Rubio-Rivas et al., 2022).

Upon completion of stool sample collection, study participants were screened via a telephone survey for re-infection episodes of COVID-19. The confirmation of re-infection with COVID-19 was double-checked through the Korea Centers for Disease Control and Prevention (KCDC). We only re-examined re-infections, focusing on the patient’s symptoms, recovery severity, and microbiome analysis. There were some limitations in collecting additional personal data with regard to vaccination status.

### Stool sample processing and DNA extraction

Stool was collected in sterile collection cups using a Para-Pak^®^ stool transport vial (Meridian Bioscience Inc., Cincinnati, Ohio, USA) and immediately sent to the research team. Stool samples were processed in the laboratory within 2 hour of arrival and stored at −80°C until analysis. Microbial DNA was extracted from approximately 200 mg of feces per sample using the FastDNA™ SPIN Kit for Soil (MP Biomedicals, Solon, Ohio, USA), according to the manufacturer’s protocol.

### Metagenomic sequencing

Polymerase chain reaction (PCR) amplification was performed using primers targeting the V3–V4 regions of the 16S rRNA gene with the extracted DNA. For bacterial amplification, the primers 341F (5′-TCGTCGGCAGCGTC-AGATGTGTATAAGAGACAG-CCTACGGGNGGCWGCAG-3′) and 805R (5′-GTCTCGTGGGCTCGGAGATGTGTATAAGAGACAGGACTACHVGGGTATCTAATCC-3′) were used. The fusion primers were constructed in the following order: P5 (P7) graft-binding, i5 (i7) index, Nextera consensus, sequencing adaptor, and target region sequence. Amplification was performed in a C1000 touch thermal cycler PCR system (Bio-Rad Laboratories, Inc., Hercules, CA, USA) under the following conditions: initial denaturation at 95°C for 3 min, followed by 25 cycles of denaturation at 95°C for 30 s, primer annealing at 55°C for 30 s, and extension at 72°C for 30 s, with a final elongation at 72°C for 5 min. The PCR product was confirmed by 1% agarose gel electrophoresis and visualized using a Gel Doc system (BioRad, Hercules, CA, USA). The amplified products were purified with the ProNex size-selective purification system (Promega, USA) with a 1:1.5 v/v ratio of sample to beads. Quality and product size were assessed on a Bioanalyzer 2100 (Agilent, Palo Alto, CA, USA) using a DNA 7500 chip. Mixed amplicons were pooled, and sequencing was carried out at CJ Bioscience (Seoul, Korea) using the Illumina MiSeq Sequencing system (Illumina, USA) with 2 × 250 bp paired-end mode according to the manufacturer’s instructions.

### Metagenome analysis

Taxonomic profiling was performed using EzBioCloud’s MTP 16S service with the PKSSU4.0 reference database. Operational Taxonomic Unit (OTU) read counts were imported into R V.4.0.3 for statistical analysis. Propensity score matching in MatchIt R package V.4.4.0 was used to select individuals from the healthy controls closest to the COVID-19 patients. The OTU read counts of the samples were rarefied to 12,746 (minimum read count among the samples). For the alpha diversity analysis, Shannon diversity and species richness were calculated. The microbial diversity of the two groups, including paired data from the same patients (early and late convalescent phase), was compared using the Wilcoxon rank sum test. We also assessed normality using Q-Q plots and the Shapiro-Wilk test (**Supplementary Figure S1**) and calculated parametric Student’s t-test results (**Supplementary Figure S2**). As for Beta diversity, the Bray-Curtis index was used to calculate microbiome dissimilarities, and principal coordinate analysis (PCoA) was carried out to visualize the dissimilarities. Permutational multivariate analysis of variance (PERMANOVA) was used to determine whether the dissimilarities were associated with certain sample parameters. Distance-based redundancy analysis (dbRDA) was conducted to assess the influence of confounding factors on the other factors. The vegan R package V.2.5-7 was used for alpha diversity, Bray-Curtis, PERMANOVA, dbRDA, and rarefying. Linear discriminant analysis effect size (LEfSe) was used to identify the microbial biomarkers associated with certain sample parameters. The MaAsLin2 R package V.1.10.0, was used to identify multivariable associations between parameters and taxonomic features.

### Statistical analysis

Categorical variables were compared using the Chi-square test and presented as number (percent). Continuous variables were expressed as mean ± standard deviation, and they were analyzed using the non-parametric Mann–Whitney U or Kruskal–Wallis test. All two-tailed *P*-values of < 0.05 were considered statistically significant. The statistical analyses were performed using SPSS Version 23.0 software (SPSS Inc., Chicago, IL, USA).

### Data and materials availability

All sequencing data is available at PRJNA950198.

## Results

### Clinical characteristics of the study populations

Stool samples from the early and late convalescent phases of COVID-19 patients (n = 58) were compared with uninfected controls (n = 116) of the COVID-19 naive group (**Supplementary Table S1**). Patients with COVID-19 were stratified into three different groups according to the WHO severity scale. Clinical symptoms of fever (45.8% in the mild group *vs*. 86.4% in the moderate group *vs.* 75.0% in the severe group, *P* = 0.011) and COVID-19-related pneumonia (12.5% *vs*. 63.6% *vs.* 100%, *P* < 0.001) were more prevalent in the moderate and severe groups than in the mild COVID-19 group. All patients with severe COVID-19 received mechanical ventilation care (0% *vs*. 0% *vs.* 100%, *P* < 0.001) and intravenous steroid therapy (0% *vs*. 36.4% *vs.* 100%, *P* < 0.001). Most patients with severe COVID-19 were treated with remdesivir (0% *vs*. 36.4% *vs.* 91.7%, *P* < 0.001) and systemic antibiotic therapy (4.2% *vs*. 54.5% *vs.* 83.3%, *P* < 0.001) (**Table 1**).

**Table 1:** Clinical characteristics of study populations.

Variables	Controls n = 116	COVID-19			*p*-value
		Mild n = 24	Moderate n = 22	Severe n = 12	
Age, years	53.6 ± 13.4	46.6 ± 17.0	60.4 ± 10.9	61.7 ± 12.6	0.002
Sex, male	52 (44.8)	9 (37.5)	12 (54.5)	5 (41.7)	0.703
BMI, kg/m^2^	23.7 ± 2.6	22.9 ± 2.8	25.2 ± 3.8	26.2 ± 3.5	0.001
Smoking history					
Never smoked	–	19 (79.2)	12 (54.5)	11 (91.7)	0.043
Current or ex-smoker	–	5 (20.8)	10 (45.5)	1 (8.3)
Comorbidity					
Hypertension	–	2 (8.3)	8 (36.4)	7 (58.3)	0.005
Diabetes	–	0 (0)	1 (4.5)	2 (16.7)	0.102
COPD	–	0 (0)	1 (4.5)	0 (0)	0.435
Cardiovascular diseases	–	0 (0)	1 (4.5)	0 (0)	0.435
Clinical manifestations					
Fever	–	11 (45.8)	19 (86.4)	9 (75.0)	0.011
Peak BT, °C	–	38.4 ± 0.8	38.2 ± 0.5	38.5 ± 0.7	0.636
Diarrhoea	–	2 (8.3)	5 (22.7)	1 (8.3)	0.304
Nausea	–	2 (8.3)	2 (9.1)	0 (0)	0.568
Vomiting	–	2 (8.3)	1 (4.5)	0 (0)	0.560
Pneumonia^*^	–	3 (12.5)	14 (63.6)	12 (100.0)	< 0.001
Treatment					
Ventilator care	–	0 (0)	0 (0)	8 (66.7)	< 0.001
Haemodialysis	–	0 (0)	0 (0)	1 (8.3)	0.142
Remdesivir	–	0 (0)	8 (36.4)	11 (91.7)	< 0.001
Steroids	–	0 (0)	8 (36.4)	12 (100.0)	< 0.001
Antibiotics	–	1 (4.2)	12 (54.5)	10 (83.3)	< 0.001
Time interval^†^, days					
Early phase	–	43.7 ± 15.0	46.2 ± 22.1	53.3 ± 16.3	0.341
Late phase	–	222.7 ± 29.5	234.8 ± 37.6	238.5 ± 16.2	0.260
Re-infection^‡^	–	3 (12.5)	1 (4.5)	1 (8.3)	0.630

Data are expressed as number (%) and mean ± standard deviation. ^*^COVID-19 related pneumonia. ^†^Time interval between stool collection and confirmation date of COVID-19. ^‡^Re-infection with B.1.1.529 variant of Severe Acute Respiratory Syndrome-Coronavirus-2.

BMI, body mass index; BT, body temperature; COPD, chronic obstructive pulmonary disease; COVID-19, coronavirus diseases 2019.

The mean time intervals from the date of first stool collection and confirmation date of COVID-19 infection in each group were 43.7 ± 15.0 days, 46.2 ± 22.1 days, and 53.3 ± 16.3 days, respectively. Late convalescent phase data at the time of the second stool sample collection were 222.7 ± 29.5 days, 234.8 ± 37.6 days, and 238.5 ± 16.2 days, respectively, from COVID-19 confirmation date.

Based on COVID-19 genomic surveillance by the Korea Centers for Disease Control and Prevention (KCDC), it was estimated that all subjects in the COVID-19 patient group were infected with either the wild-type or B.1.617.2 (delta) variants of SARS-CoV-2. According to a survey conducted in July 2022, when the B.1.1.529 (Omicron) variant of the SARS-CoV-2 outbreak in South Korea decreased, only five patients among the study populations were re-infected with the B.1.1.529 variant of SARS-CoV-2.

### Relative stool microbiome abundance and distribution between COVID-19 patients and COVID-19 naive controls

**Figure 1:**
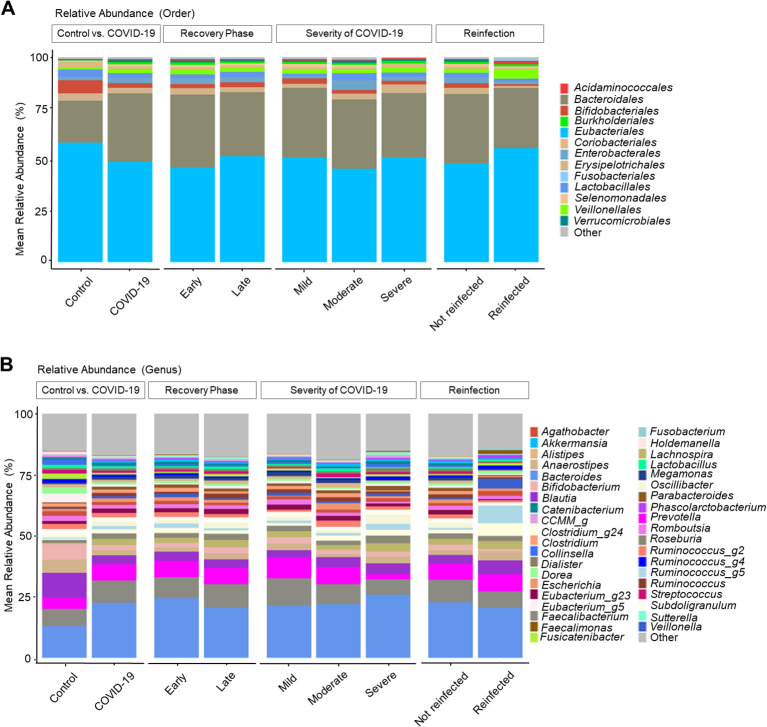
Taxonomic profiles of COVID-19 and uninfected healthy controls. **(A)** Order level. **(B)** Genus level.

The gut microbiome changes at the order and genus levels in COVID-19 patients compared with controls are depicted in **Figure 1**. Specifically, the mean abundance of *Eubacteriales* (58.3% in the control group vs. 49.0% in the COVID-19 group, *P* < 0.001) and *Bifidobacteriales* (6.5% vs. 2.2%, *P* < 0.001) decreased, while *Bacteroidales* (20.5% vs. 33.4%, *P* < 0.001) and *Burkholderiales* (0.2% vs. 1.0%, *P* < 0.001) increased in the COVID-19 group compared to the controls (**Figure 1A**). At the genus level, the mean abundance of *Bacteroides* still showed a significant increase (13.1% vs. 22.6%, *P* < 0.001) in the COVID-19 group compared to the controls. Meanwhile, *Blautia* (10.4% vs. 3.7%, *P* < 0.001), *Bifidobacterium* (6.5% vs. 2.2%, *P* < 0.001), and *Lactobacillus* (1.4% vs. 0.8%, *P* = 0.002) displayed a decreasing trend in the COVID-19 group compared to the controls (**Figure 1B**).

Analysis by recovery time revealed an increase in *Eubacteriales* and *Bifidobacteriales*, along with a decrease in *Bacteroidales* and *Burkholderiales* in the late convalescent phases. The overall microbiome composition exhibited a recovery tendency similar to that of the uninfected group; however, full recovery was not observed.

Regarding the COVID-19 severity, the moderate and severe groups showed decreased levels of *Bifidobacterium* and increased *Bacteroides* compared to the mild group. Additionally, *Prevotella* and *Faecalibacterium* displayed a decreasing trend with the increased severity of COVID-19 infection.

### Stool microbiome diversity and similarity in COVID-19 patients compared to naive controls

**Figure 2:**
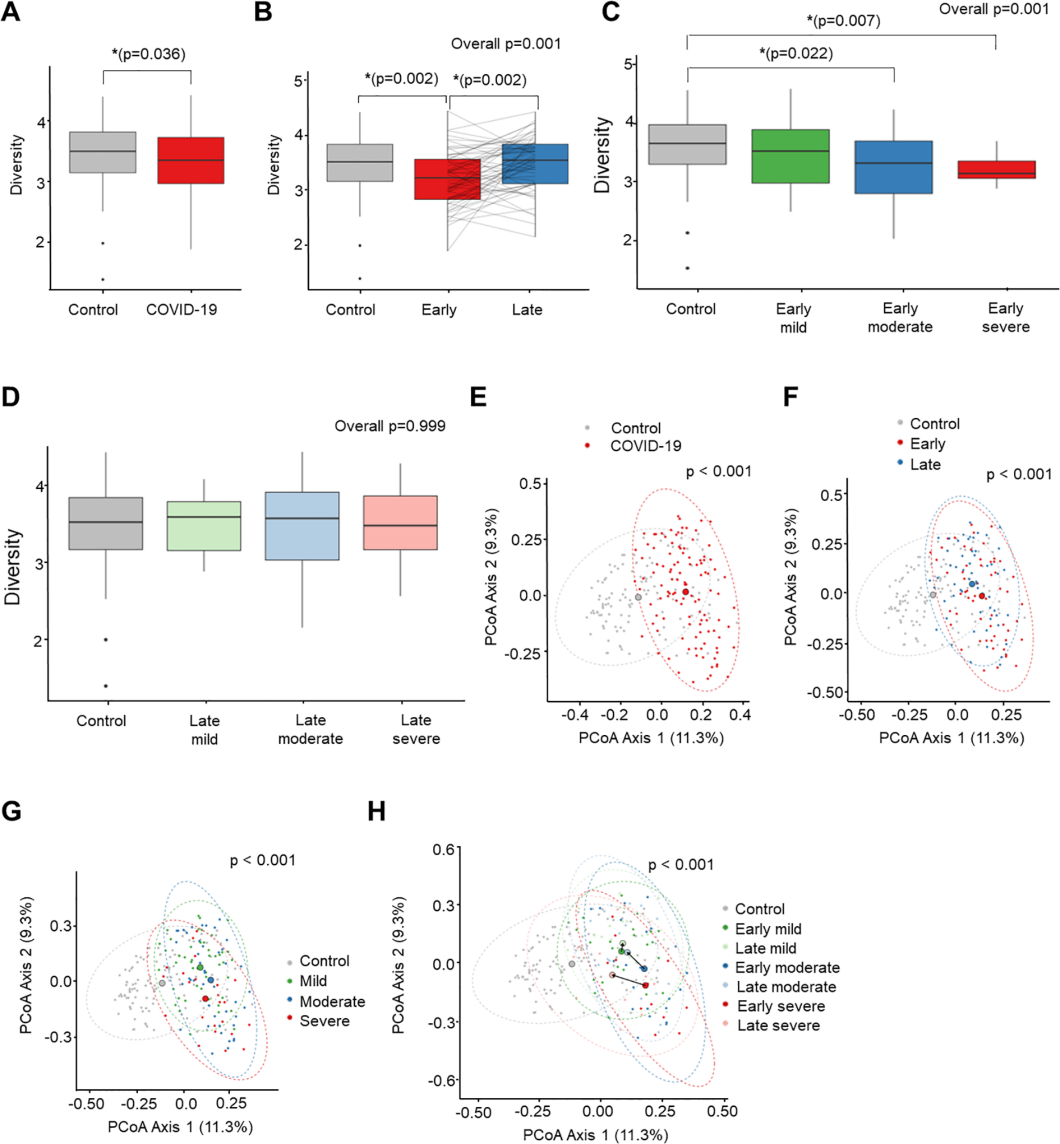
Comparison of alpha diversity (Shannon index) between COVID-19 and uninfected healthy controls **(A-D)**. PCoA analysis based on Bray-Curtis dissimilarity distance between COVID-19 and uninfected healthy controls **(E-H)**. **(A)** Control *vs.* COVID-19. **(B)** Recovery time analysis. **(C)** Severity analysis in early convalescent phase. **(D)** Severity analysis in late convalescent phase. **(E)** Control *vs.* COVID-19. **(F)** Recovery time analysis. **(G)** Severity analysis. **(H)** Recovery time and severity analysis. *P < 0.05.

The mean community richness and microbial diversity were significantly lower in the COVID-19 group compared to the control group (*P* = 0.036), as indicated by the Shannon diversity index (**Figure 2A**). Additionally, alpha diversity value was significantly lower in the early convalescent phase of COVID-19 compared to the control group (*P* = 0.002), with a near recovery in the late convalescent phase compared with the early phase of COVID-19 infection (*P* = 0.002) (**Figure 2B**). Analysis based on the severity of COVID-19 during the early phase of infection and the corresponding control stool samples revealed a decrease in Shannon index as the severity of COVID-19 infection increased (**Figure 2C**). Notably, significant differences in microbiome community were observed between the control and the moderate (*P* = 0.022), and the severe groups (*P* = 0.007) during the early phase. However, these severity-associated differences in the microbiome community were absent during the late convalescent phase (**Figure 2D**).

Next, we conducted compositional analyses to assess differences among microbial communities. The Principal Coordinates Analysis (PCoA) of Bray-Curtis distance revealed a significant distinction in the overall microbial composition between patients with COVID-19 and uninfected controls (*P* < 0.001) (**Figure 2E**). Comparing the overall microbiome composition between the early and late convalescent phases showed no significant difference in beta diversity in the PCoA analysis (early *vs.* late, *P* = 0.123) (**Figure 2F**). However, subgroup analysis by disease severity revealed major alterations between the groups (mild *vs.* moderate, *P* = 0.018; mild *vs.* severe, *P* = 0.001; moderate *vs.* severe, *P* = 0.043) (**Figure 2G**). Furthermore, significant compositional differences in the distance between the early and late phases were observed according to severity, particularly in the moderate and severe groups (early phase *vs*. late phase: mild group, *P* = 0.130; moderate group, *P* = 0.027; severe group, *P* = 0.010) (**Figure 2H**). Pairwise PERMANOVA *P*-values for each group are presented in **Supplementary Table S2**.

### Linear discriminant analysis on the effect size of the gut microbiome

**Figure 3:**
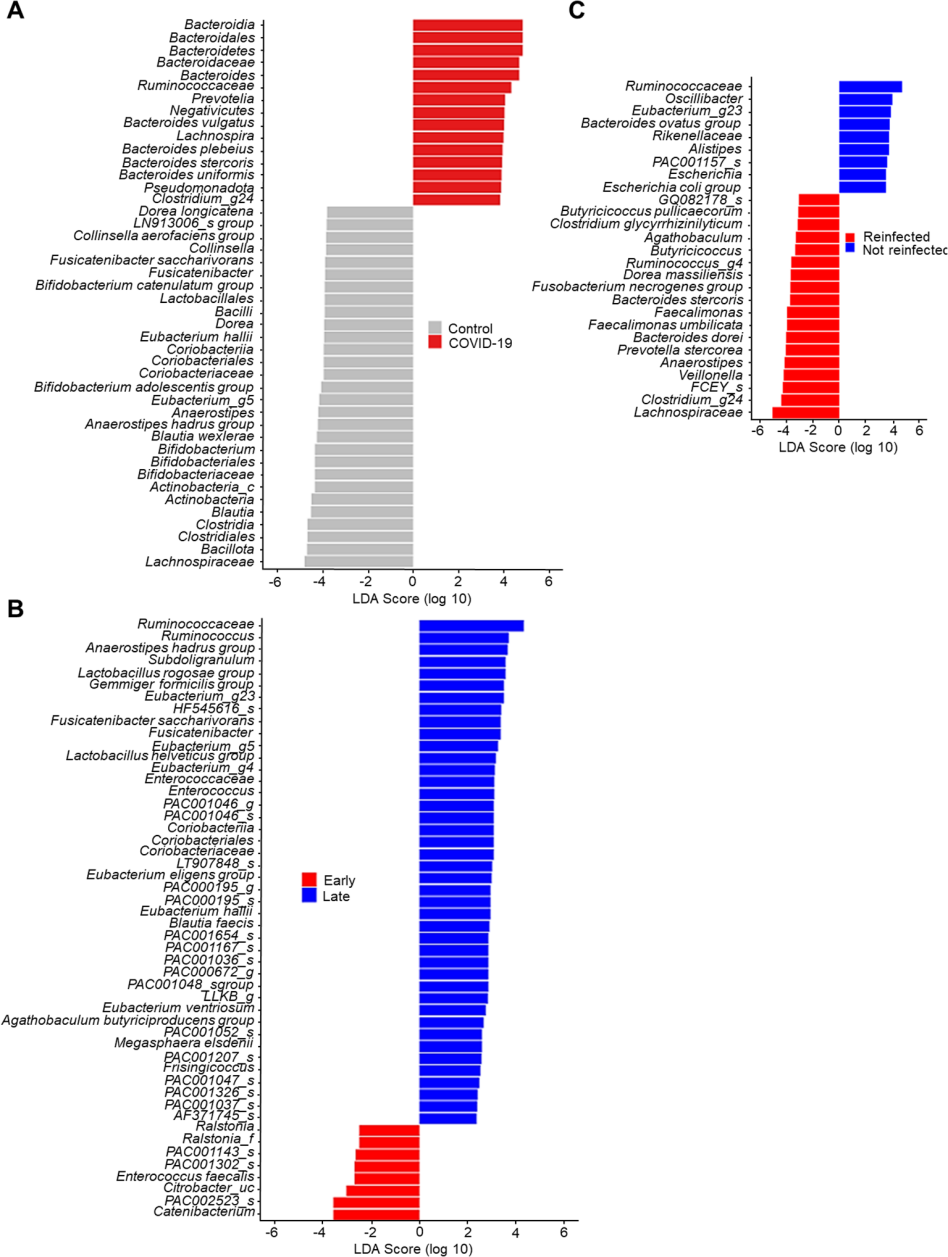
Linear discriminant analysis effect size of stool microbiome. **(A)** Control *vs.* COVID-19. **(B)** Early convalescent phase *vs.* Lase convalescent phase. **(C)** Wild type or delta variant of SARS-CoV-2 infection *vs.* Omicron variant of SARS-CoV-2 re-infection.

In **Figure 3**, the lengths of the bar columns represent the linear discriminant analysis (LDA) score, which indicates significant differences in bacterial abundances of the stool microbiome between the two groups. LDA scores (log10) > 2 and *P*-value < 0.05 are listed in **Figure 3A** displays the microbial taxa with significant differences between COVID-19 infection (Positive score) and uninfected controls (Negative score). In the control group, *Bacillota* (genus *Blautia* & *Anaerostipes*) and *Actinomycetota* (genus *Bifidobacterium*), bacteria easily found in probiotic products or producing butyrate, were dominant. Conversely, as described earlier, *Bacteroidota* and *Pseudomonadota* were dominant in the COVID-19 group.

The LDA scores of the recovery time (**Figure 3B**) indicate that *Enterococcus faecalis*, bacteria known for their antibiotic resistance, were dominant in the early convalescent phase of COVID-19 (Negative score). In contrast, several butyrate-producing bacteria such as *Subdoligranulum, Anaerostipes*, and *Eubacterium* became dominant in the late convalescent phase of COVID-19 (Positive score).

In our cohort, five patients were re-infected with the B.1.1.529 (Omicron) variant of SARS-CoV-2. Therefore, we conducted comparative microbiome analyses between those infected once versus twice with SARS-CoV-2. Compared to patients infected with either the wild type or the delta variant of SARS-CoV-2 (negative score), those re-infected with the B.1.1.529 variant (positive score) were found to have *Lachnospiraceae* and *Faecalimonas umbilicata* as the dominant species (**Figure 3C**).

### Minimal influence of antibiotic use and age on WHO severity’s effect on gut microbiome

To determine whether the effect of the WHO severity scale on the microbiome was independent of other factors such as antibiotic use and age, we conducted multivariable analyses using dbRDA and MaAsLin2.

**Figure 4:**
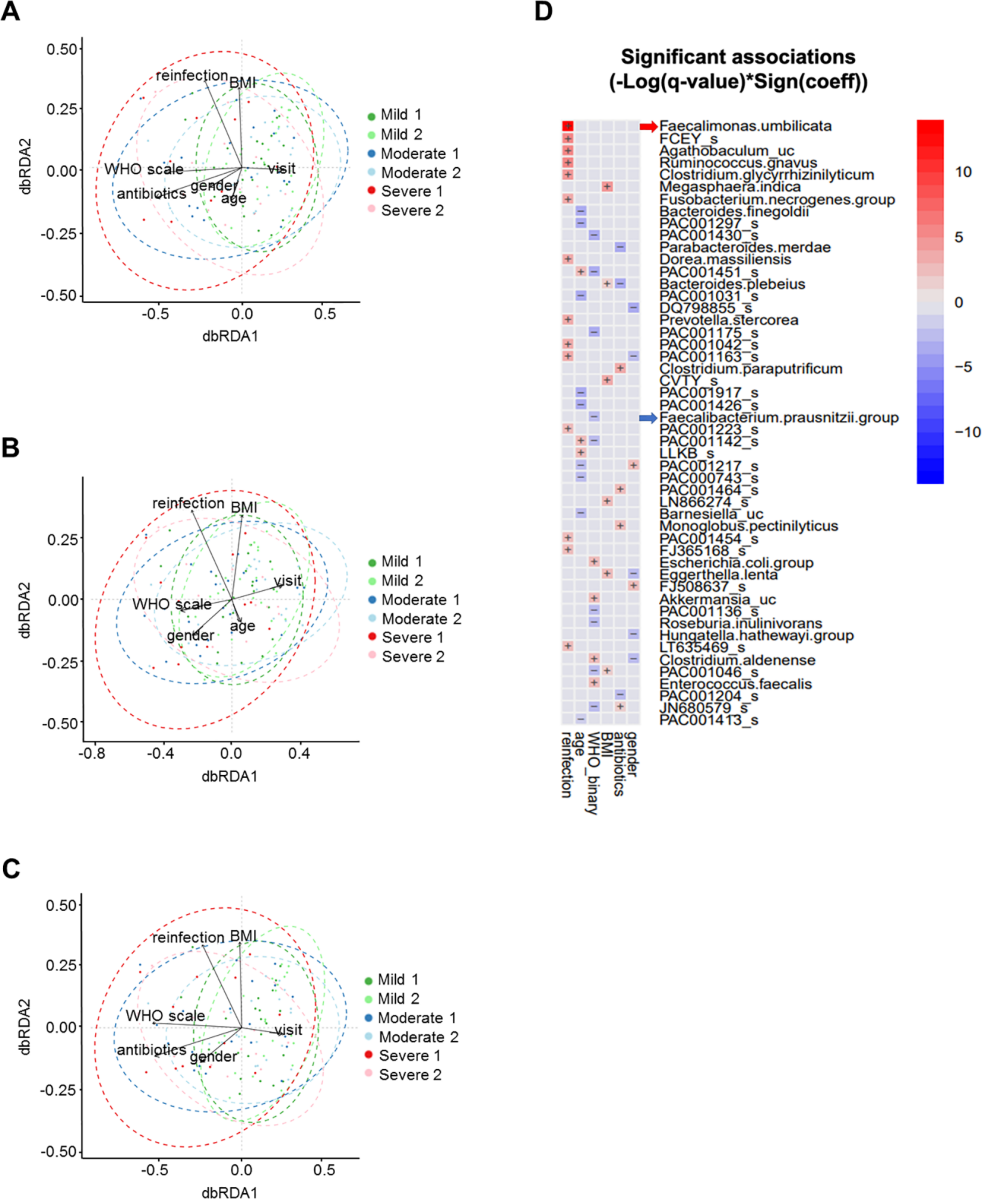
Multivariable analyses of stool microbiome. **(A)** dbRDA with WHO severity scale, antibiotic use, age, time of stool collection, gender, SARS-CoV-2 reinfection, and BMI. **(B)** dbRDA with antibiotic use partialled out. **(C)** dbRDA with age partialled out. **(D)** MaAsLin2 heatmap showing stool microbial operational taxonomic units with significant association with clinical variables.

We constructed a dbRDA model to assess the impact of the WHO severity scale on overall microbial composition, measured by Bray-Curtis distance, while controlling for variables including antibiotic use, age, time of stool collection, gender, SARS-CoV-2 reinfection, and BMI (**Figure 4A**; **Table 2**). We used the by=“margin” option in the permutation-based ANOVA test to calculate marginal effects, ensuring that the order of variables did not bias the results. The WHO severity scale had the largest impact on microbial composition (Sum of Sqs = 0.686, *P* = 0.006), while antibiotic use showed a relatively smaller but still significant effect (Sum of Sqs = 0.545, *P* = 0.008). Age had an even smaller effect, which was not statistically significant (Sum of Sqs = 0.439, *P* = 0.068). To further assess the effect of the WHO severity scale, we partialled out antibiotic use and age from the dbRDA model, and the WHO severity scale remained significant in both cases (**Figures 4B, C**; **Table 2**).

**Table 2:** Permutation-based ANOVA for dbRDA to test the effect of confounding clinical factors and their interactions on stool microbiome composition.

Partialled out variables	Clinical variables	df	Sum of Sqs	F. model	R^2^	*p*-value
None	Antibiotic use	1	0.545	1.737	0.014	0.008
	Age	1	0.439	1.398	0.012	0.068
	WHO severity scale	1	0.686	2.185	0.018	0.006
	Re-infection	1	0.638	2.033	0.017	0.003
	Stool collection time	1	0.426	1.358	0.011	0.066
	Gender	1	0.469	1.494	0.012	0.041
	BMI	1	0.530	1.688	0.014	0.018
Antibiotic use	Age	1	0.439	1.398	0.012	0.076
	WHO severity scale	1	0.686	2.185	0.018	0.001
	Re-infection	1	0.638	2.033	0.017	0.003
	Stool collection time	1	0.426	1.358	0.011	0.078
	Gender	1	0.469	1.494	0.013	0.054
	BMI	1	0.530	1.688	0.014	0.022
Age	Antibiotic use	1	0.545	1.737	0.015	0.018
	WHO severity scale	1	0.686	2.185	0.018	0.001
	Re-infection	1	0.638	2.033	0.017	0.007
	Stool collection time	1	0.426	1.358	0.011	0.095
	Gender	1	0.469	1.494	0.013	0.050
	BMI	1	0.530	1.688	0.014	0.019

df, degree of freedom; Sqs, squares; BMI, body mass index; WHO, World Health Organization.

In addition, we performed MaAsLin2 analysis to examine multivariable associations between individual microbial taxonomic features and clinical variables, including gender, antibiotic use, BMI, WHO severity scale, age, and SARS-CoV-2 reinfection. This analysis identified microbial taxa that were specifically associated with the WHO severity scale, including *Faecalibacterium prausnitzii* (**Figure 4D**).

These analyses indicate that the WHO severity scale significantly influences microbial composition, with effects independent of antibiotic use and age.

## Discussion

The results of this study indicate that SARS-CoV-2 infection is likely associated with changes in the stool microbiome compared to the uninfected controls selected with propensity-matched scoring analyses. Specifically, our results suggest that SARS-CoV-2 infection leads to a decreased abundance of symbionts and an increased proportion of opportunistic pathogens. Seven months after SARS-CoV-2 infection, the gut microbiome community became more similar to the baseline composition. However, the extent of recovery was somewhat limited, highlighting the persistent effects of respiratory viral infection on the gut microbiota community.

We found a significant reduction in microbiome diversity in patients with severe symptoms. Moreover, we and others (Donati Zeppa et al., 2020; Shi et al., 2021) found that *Faecalibacterium* decreased as the disease severity increased. However, we did not detect an increased abundance of *Clostridium* or *Coprobacillus* in the severe COVID-19 group as reported in other studies (Donati Zeppa et al., 2020; Shi et al., 2021). Importantly, our study confirmed that the degree of gut microbiota alteration was higher in the severe group, even after considering the impact of antibiotic use and age. These results support the notion that gut microbiota diversity is negatively correlated with COVID-19 severity.

The gut microbiota composition undergoes significant changes upon SARS-CoV-2 infection, likely due to the gut-lung axis interaction as reported following respiratory infections such as influenza, respiratory syncytial virus, or chronic obstructive pulmonary diseases (Keely et al., 2012; Hanada et al., 2018; Yildiz et al., 2018; Gu et al., 2020). For example, the progression of pneumonia and acute respiratory distress syndrome triggered by respiratory infections can impact the host immune system, leading to alterations in the gut microbiome. Conversely, this interaction involves both endotoxins and metabolites from the gut microbiome, influencing the immune response of the lung. The disrupted immune-gut homeostasis may further affect disease progression due to heightened inflammatory responses (Dhar and Mohanty, 2020). ACE2, a receptor for SARS-CoV-2, regulates amino acid transport in the intestine, playing a critical role in modulating innate immunity (Perlot and Penninger, 2013). Thus, SARS-CoV-2 infection may also influence the gut microbiome through the ACE2 activity-dependent modulation of host immunity.

Our study revealed that several butyrate-producing bacteria, such as *Oscillibacter, Subdoligranulum*, and *Anaerostipes* were abundantly present in late convalescent COVID-19 patients compared to early recovered phase patients. This finding aligns with the observation that patients with severe COVID-19 often exhibit impaired production of bacterial metabolites such as short-chain fatty acids (SCFAs), which include butyrate (Zhang et al., 2022). Given that SCFAs possess anti-inflammatory properties, the reduced abundance of butyrate-producing bacteria may have contributed to the severity of COVID-19. Although it was not a significant difference in our data analysis due to an insufficient sample size; however, other literature indicates that it is also related to the patient’s hospitalization duration and the severity of respiratory infections (Haak et al., 2018; Kullberg et al., 2024).

Stool samples were collected post-recovery, allowing us to detect microbiome changes in patients re-infected with SARS-CoV-2. In the re-infected group, *Lachnospiraceae* and *Faecalimonas umbilicata* were dominant bacteria compared to the one-time infected group. While gut microbiota composition alone may not be the sole determinant of re-infection, it raises intriguing questions about the potential role of dysbiosis in predisposing individuals to re-infection. Exploring whether individuals with incomplete recovery of their microbiome community are at a higher risk of re-infection could provide valuable insights, especially in the context of seasonal flu or endemic SARS-CoV-2. Further comparative studies are needed to help validate this hypothesis.

While the severity of COVID-19 has diminished compared to the initial virulence of the SARS-CoV-2 virus (Ciceri et al., 2020; Lan et al., 2021) and has transitioned from a pandemic to an endemic state, identifying high-risk groups remains crucial for better preparedness for future pandemics. Our findings offer a potential pathway for identifying biomarkers that could pinpoint dominant microbiome species linked to disease severity and the likelihood of re-infection.

We also note that this study has several limitations. First, we did not conduct live viral culture or immune correlate analyses alongside microbiome analyses. Secondly, stool samples from deceased COVID-19 patients (the most severe group) were not included in our analysis. Additionally, the sample size of moderate and severe COVID-19 patients was relatively small, underscoring the need for larger-scale studies. Thirdly, the majority of our subjects were infected with wild-type and delta variant viruses, and data from individuals infected with omicron and their sub-lineage variants were not included. The Korea Disease Control and Prevention Agency confirmed the SARS-CoV-2 mutation pattern through sample surveillance, and divided the epidemic period based on the week in which more than 50% of the weekly variant virus tests were detected. The period when the samples were collected coincides with the period when wild type SARS-CoV-2 and delta variant were dominant. Lastly, we did not account for other factors that likely influence the gut microbiome, such as diet, lifestyle, eating habits, and living environment.

Despite these limitations, our study holds significance for several reasons. Firstly, we recruited a larger number of subjects compared to previously published studies. Secondly, we utilized follow-up data, including samples collected at seven-month post-recovery, for our analyses. We successfully identified bacteria that could serve as indicators of severe COVID-19, laying the groundwork for future predictive modeling efforts and mechanistic studies using preclinical models. Furthermore, we demonstrated that even after accounting for the effects of antibiotics and age, the severity of COVID-19 remained significantly associated with changes in microbial communities. Lastly, despite its correlative nature, our data suggest the potential utility of the microbiome as a biomarker for assessing susceptibility to re-infection.

In summary, our study reveals distinct differences in the stool microbiome composition between COVID-19 patients and uninfected controls, particularly evident during the early convalescent phase, with pronounced variations observed in the severe disease group. Notably, the severity of COVID-19 infection emerged as the most influential clinical factor in shaping gut dysbiosis, surpassing even the impact of antibiotic usage. These findings underscore the potential of predominant opportunistic microorganisms as biomarkers for assessing the severity of respiratory infectious diseases.

## Data Availability

The datasets presented in this study can be found in online repositories. The names of the repository/repositories and accession number(s) can be found in the article/**Supplementary Material**.
