# Supplementary figures and images for: Altered gut microbiome in convalescent patients with coronavirus disease 2019
Source: Front Cell Infect Microbiol. 2024 Nov 28;14:1455295. doi: 10.3389/fcimb.2024.1455295 (PMC11634865; doi:10.3389/fcimb.2024.1455295)

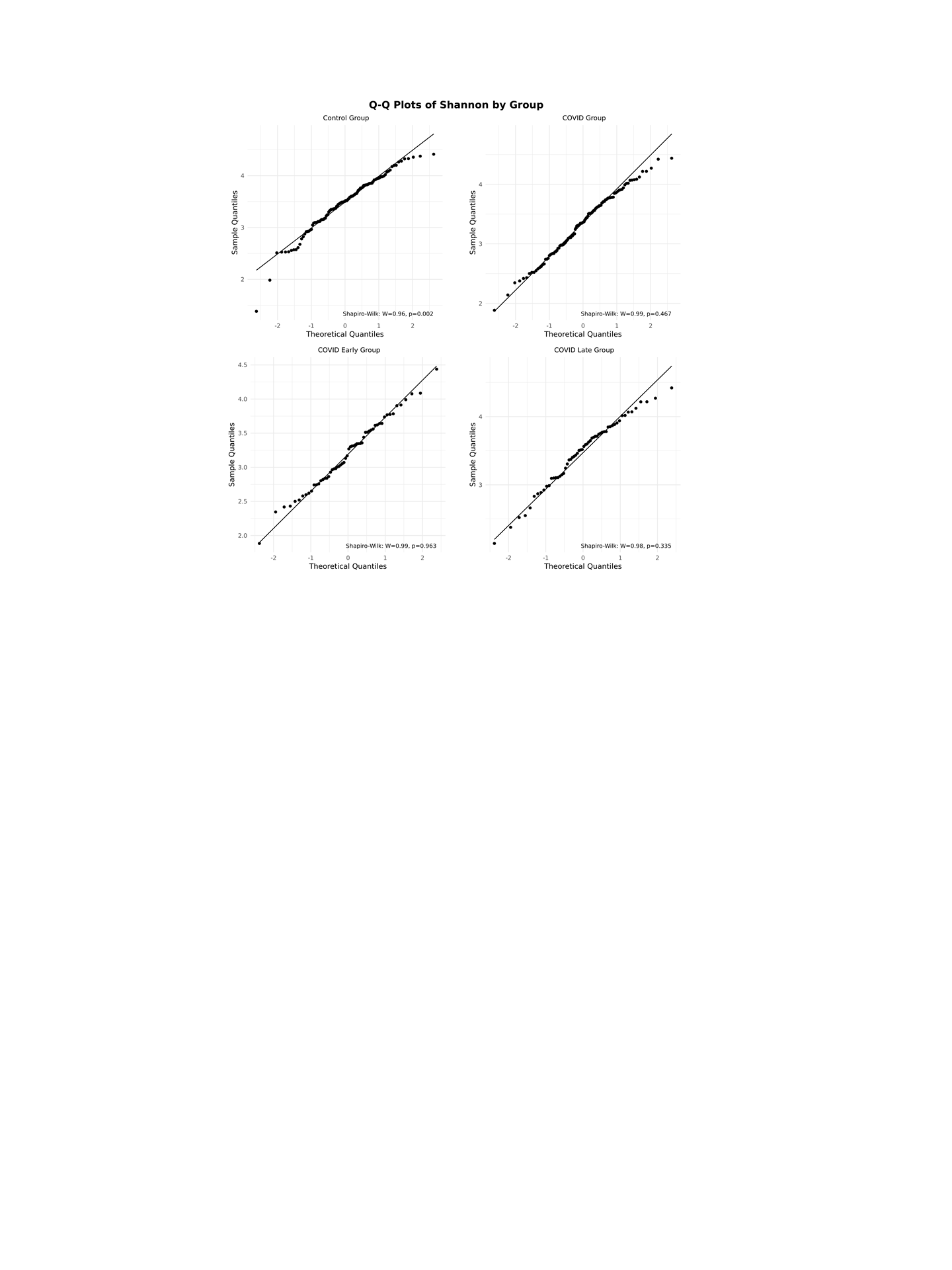

Supplement: Supplementary file 1 [file Image1.tif]

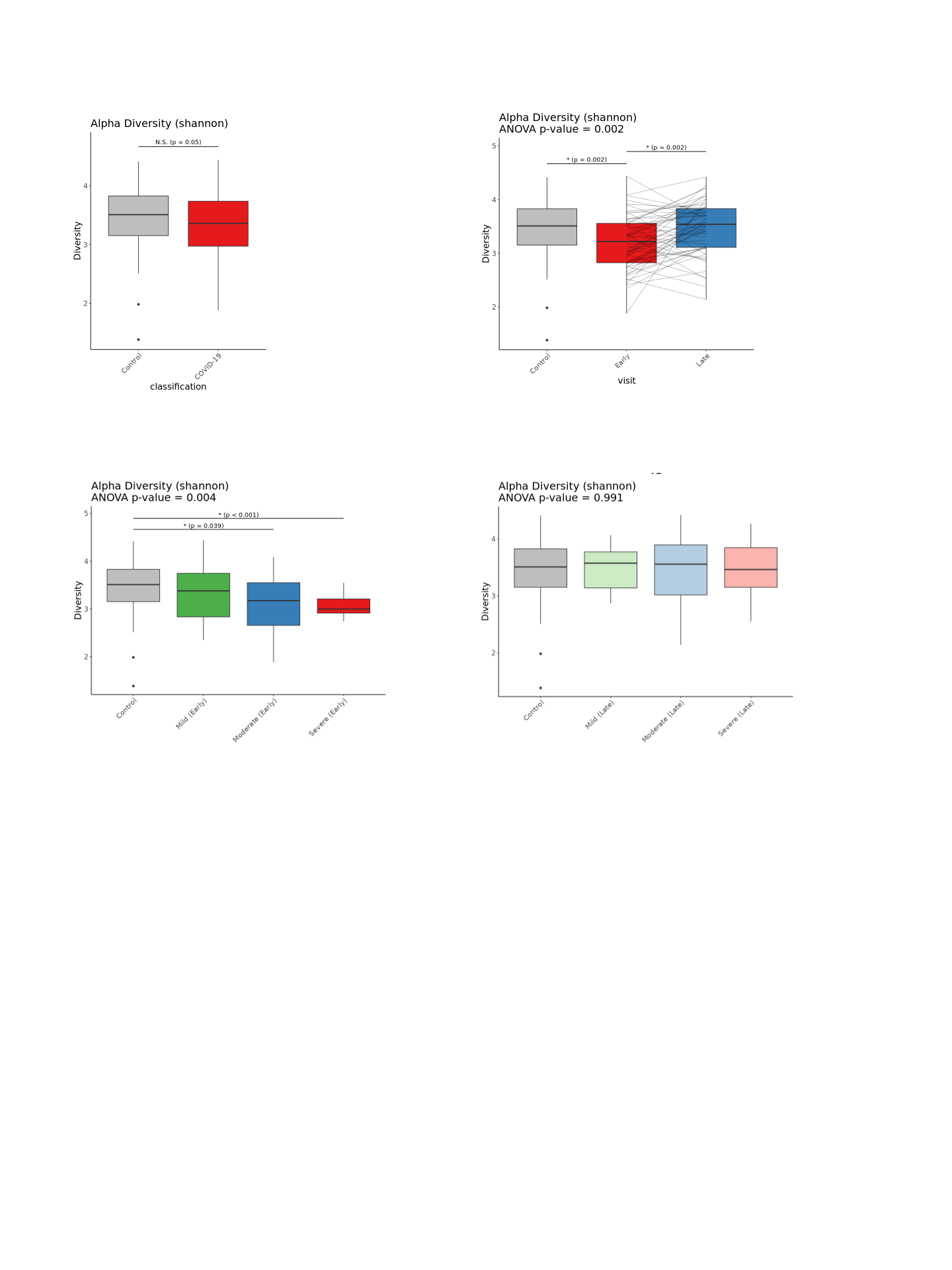

Supplement: Supplementary file 2 [file Image2.tif]
