# Supplementary material for: Altered gut microbiome in convalescent patients with coronavirus disease 2019
Source: Front Cell Infect Microbiol. 2024 Nov 28;14:1455295. doi: 10.3389/fcimb.2024.1455295 (PMC11634865; doi:10.3389/fcimb.2024.1455295)
Supplement: Supplementary file 3 [file Table1.docx]

**Supplementary Figure S1. Q-Q plots with Shapiro-Wilk scores (Shannon index)**

**Supplementary Figure S2. Shannon index with Student’s t-test and ANOVA**

**Supplementary Table S1. Demographic characteristics of healthy controls and COVID-19 patients.**

| **Variables** | **Non-COVID-19**  **controls**  **n = 116** | **COVID-19**  **n = 58** | ***p*-value** |
| --- | --- | --- | --- |
| Age, years | 53.6 ± 13.4 | 55.0 ± 15.6 | 0.563 |
| Sex, male | 52 (44.8) | 26 (44.8%) | > 0.999 |
| BMI, kg/m^2^ | 23.7 ± 2.6 | 24.4 ± 3.6 | 0.143 |

Data are expressed by number (%) and mean ± standard deviation.

Abbreviations: BMI, body mass index; COVID-19, coronavirus diseases 2019

**Supplementary Table S2. Permutational multivariate analysis of variance (PERMANOVA) *p*-value of each paired groups.**

| ***vs.*** | | **COVID-19** | | | | | |
| --- | --- | --- | --- | --- | --- | --- | --- |
| **Control** | | < 0.001 | | | | | |
| ***vs.*** | | **Early** | | | **Late** | | |
| **Control** | | 0.001 | | | 0.001 | | |
| **Early** | | - | | | 0.123 | | |
| ***vs.*** | | **Mild** | | **Moderate** | | **Severe** | |
| **Control** | | 0.001 | | 0.001 | | 0.001 | |
| **Mild** | | - | | 0.018 | | 0.001 | |
| **Moderate** | | - | | - | | 0.043 | |
| **Severe** | | - | | - | | - | |
| ***vs.*** | | **Mild** | | **Moderate** | | **Severe** | |
|  |  | **Early** | **Late** | **Early** | **Late** | **Early** | **Late** |
| **Control** | | 0.003 | 0.003 | 0.003 | 0.003 | 0.003 | 0.003 |
| **Mild** | **Early** | - | 0.130 | 0.081 | 0.515 | 0.019 | 0.199 |
|  | **Late** | - | - | 0.025 | 0.589 | 0.003 | 0.081 |
| **Moderate** | **Early** | - | - | - | 0.027 | 0.515 | 0.140 |
|  | **Late** | - | - | - | - | 0.061 | 0.087 |
| **Severe** | **Early** | - | - | - | - | - | 0.010 |
|  | **Late** | - | - | - | - |  | - |

The stool samples of non-COVID-19 healthy controls were collected before the COVID-19 pandemic.

The stools of the early convalescent phase were collected at the end of quarantine or hospitalization, and the late phase stool samples were collected six months after the COVID-19 confirmation date.

The severity of disease was stratified as mild (severity score, l; no limitation of activities, and 2; limitation of activities), moderate (3; hospitalized with no oxygen therapy, and 4; oxygen therapy by mask or nasal prongs), and severe (5: non-invasive mechanical ventilation or high-flow oxygen therapy, 6: mechanical ventilation, and 7: mechanical ventilation with renal replacement therapy or Extracorporeal Membrane Oxygenation) according to the WHO ordinal severity scale based on the clinical and respiratory status of COVID-19 patients.

Abbreviations: COVID-19, coronavirus diseases 2019
